# Prevalence and risk factors of primary open-angle glaucoma in a city of Eastern China: a population-based study in Pudong New District, Shanghai

**DOI:** 10.1186/s12886-015-0124-x

**Published:** 2015-10-13

**Authors:** Jiangnan He, Haidong Zou, Richard K. Lee, Xiaowei Tong, Wenli Tang, Yi Zhang, Rong Zhao, Ling Ge

**Affiliations:** Shanghai Eye Disease Prevention and Treatment Center, No. 380, Kangding Road, Jingan, Shanghai, 200040 China; Shanghai General Hospital, Shanghai Jiao Tong University, No. 100 Haining Road, Shanghai, 200080 China; Bascom Palmer Eye Institute, 900 N.W. 17th Street, Miami, FL 33136 USA; Huamu Community Health Service Center, No. 96 Yulan Road, Shanghai, Pudong New District 201204 China; Shanghai Shenkang Hospital Development Center, No. 2 Kangding Road, Jingan, Shanghai, 200040 China

**Keywords:** Population-based, Prevalence, Primary open-angle glaucoma, Risk factors

## Abstract

**Background:**

The aim of the present study was to investigate the prevalence and characteristics of primary open-angle glaucoma among the urban population of Pudong New District, Shanghai.

**Methods:**

Three residents’ committees were randomly selected from Pudong New District, and residents aged 50 and older were screened for primary open-angle glaucoma (POAG) from March to April 2011. In remote screening, the tests on visual acuity, refraction, intraocular pressure (IOP), and the photographs of anterior segment and fundus were used to identify POAG suspect. The suspected subjects were then reexamined with the tests on IOP, gonioscopy, Humphrey visual field test, and retinal nerve fiber layer (RNFL) thickness by optical coherence tomography (OCT). POAG was diagnosed according to the criteria defined by International Society for Geographic and Epidemiological Ophthalmology (ISGEO). Finally, POAG risk factors were evaluated using logistic regression analysis.

**Results:**

A total of 2528 citizens out of 3,146 eligible residents (80.36 %) participated in this study. Among the citizens, 72 were diagnosed to have POAG, giving the crude prevalence rate of 2.85 % (95 % CI:2.20 %–3.50 %) in general and age- and gender-adjusted prevalence rate of 2.8 % (95 % CI: 2.78 %–2.83 %). Among the 72 POAG patients, only 22 cases had IOP exceeding 21 mmHg while other 50 cases had IOP of 21 mmHg or less; nine cases had one eye blind (12.5 %). Intriguingly, only eight cases (11.11 %) had been diagnosed with POAG before this screening.

**Conclusions:**

More efforts are required for early screening and education on POAG in communities, especially in a POAG high-risk population.

## Background

As the second leading cause of blindness in the world, glaucoma is defined as a group of ocular disorders characterized by optic neuropathy associated with rising pathological intraocular pressure (IOP). Untreated glaucoma can permanently damage the optic nerve and result in visual field defects, which can lead to blindness. Once lost, vision normally cannot be recovered [1]. Recent population-based studies worldwide have reported that primary open-angle glaucoma (POAG) is the main type of glaucoma and the prevalence rates range from 0.5 to 7.0 % in adults aged 40 and older [2]. However, the rates vary with races and regions [3].

Population-based screening for POAG has been carried out in some regions of China, including four locations in the north (Inner Mongolia [4], Beijing [5], Handan [6], and Harbin [7]), one in the south (Guangzhou [8]), and one in the southwest (Yunnan [9]). In the east of China, especially in Shanghai with a higher percentage of aging population, however, no screening has been performed. Therefore, in this study, residents aged 50 and older in Pudong New District of Shanghai were telemedically screened to reduce high costs of labor, services, equipment, and supplies involved in complicated POAG diagnosis. The present study reported the prevalence and risk factors of POAG in an urban adult population aged 50 and older in eastern China according to the definitions of ISGEO.

## Methods

### Investigation place and targets

This study selected Huamu community in Pudong New District of Shanghai as a place for investigation. This community has a history of more than 30 years and is home to a stable population. The social and economical levels in Huamu community have reached the average level of Shanghai.

The investigation was in accordance with the Declaration of Helsinki and had passed the examination of the Ethics Committee of Shanghai General Hospital Affiliated to Shanghai Jiaotong University (registration number: 2010 K059). In addition, all subjects had signed informed consents before being examined.

### Sample size calculation

According to an investigation published in China after 2000, the overall glaucoma prevalence rate of people aged 50 and older is 2.07–3.08 % [8]. The required sample size by simple random sampling is calculated using the following formula: *n* = *Z*^2^(*p*) (1 – *p*)/*B*^2^, where *p* is the estimated prevalence, *B* is the presupposition error, and *Z* = 1.96 (at 95 % confidence interval). Assuming that *p* = 3.0 %, *B* = 3.0 % × 0.25 = 0.0075, and *Z* = 1.96, then *n* = 1988. Suppose that the sample effect coefficient of this study is 1.1 and the estimated examination rate is 90 %. The calculated number of samples is 2430.

The targets of this study were the inhabitants aged 50 and older by December 31, 2010, in Huamu community. Three of eight residents’ committees in this community were selected using the random cluster sampling method. Huamu Community Health Service Center obtained the list of inhabitants from the household register office of the local police station, and the selected residents’ committees verified the resident population. The inclusion criterion was people aged 50 and older by the end of 2010 who had been residing in the areas of the three residents’ committees for more than 6 months. The exclusion criterion was people who were registered but failed the verification or who were registered but had already died. According to the preceding criteria, a total of 3146 people aged 50 and older were living in these areas, meeting the sample size requirement.

### Investigation procedure

This research adopted remote screening in the community in combination with reexamination and diagnosis at a tertiary eye hospital. Community-based remote screening was conducted from March to April 2011. The investigation team consisted of two trained ophthalmologists (eye disease diagnosis), an optometrist (refractive correction and IOP measurement), and two technicians (anterior segment photography and fundus photography) from Shanghai Eye Disease Prevention and Treatment Center (a tertiary eye hospital), as well as two nurses (visual acuity examination and recording) and an ophthalmologist (onsite quality control and coordination) from Huamu Community Health Service Center. The project was led by an epidemiologist on prevention of blindness from Shanghai General Hospital Affiliated to Shanghai Jiaotong University. Huamu Community Health Service Center was responsible for work organization, and the residents’ committees worked in collaboration with the center. The center notified each residents’ committee 2 days before the investigation, and then the committee notified the subjects. When the investigation approached its end, the committee telephoned the subjects who had not been checked for an examination. The preliminary remote screening was conducted at Huamu Community Health Service Center, which was 5- to 15-min walk from the residential places of the subjects. The survey was conducted to collect personal information according to appointments after the subjects’ identities (IDs) were checked. Then, remote screening was performed, including visual acuity examination, refractive correction, IOP measurement, digital anterior eye structure photography by a slit lamp, and digital fundus photography. After the examination, the subjects gave their satisfaction rates on the screening procedure. On the day of the investigation, the written or digital information collected during remote screening was input into the self-made database. Then, the information was transferred to Shanghai Eye Disease Prevention and Treatment Center through the dedicated network for Shanghai eye disease prevention. After that, two associate chief physicians clinically experienced in glaucoma diagnosis for many years viewed the photographs and checked the information.

**Fig. 1:**
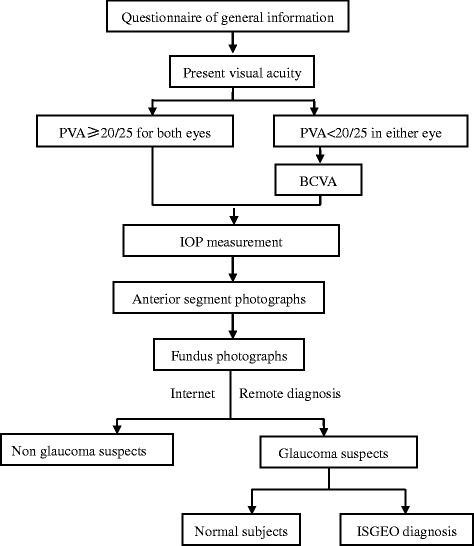
The flow chart of investigation procedure

The investigation team made an appointment for a further reexamination at Shanghai Eye Disease Prevention and Treatment Center for glaucoma suspects after the preliminary check. The reexaminations included IOP recheck, gonioscopy, perimetry test by Humphrey automated perimeter, and retinal nerve fiber layer (RNFL) thickness measurement by optical coherence tomography (OCT). Two associate chief physicians expert in clinical glaucoma diagnosis with experience of many years finally determined POAG. A flow chart of investigation procedure is shown in Fig. 1.

### Investigation methods

A standard questionnaire was administered by a trained nurse to collect details about age, gender, occupation, education degree, previous eye diseases and systemic diseases, medical treatment history, family history of diseases, and the information related to past diagnosis for eye diseases.

Visual acuity was measured using a standard illuminated LogMAR (minimum angle of resolution) E chart (Precision Vision, IL, USA), and the presenting visual acuity with habitual correction was recorded. For those with presenting visual acuity of less than 20/25 in either eye, the noncycloplegic refraction was measured using an auto-refractometer (Topcon KR.8000, Japan), and then the best corrected visual acuity (BCVA) was recorded. Atrophy of eyeball or loss of eyes was recorded as lack of vision.

In terms of IOP measurement, noncontact tonometer (Topcon CT.80, Japan) was used for both preliminary screening and reexamination. The average value of three measurements was recorded for each eye. If a subject had atrophy of eyeball or loss of eyes or was non-cooperative, the IOPs were considered not measurable.

Anterior segment of the subjects was digitally photographed using a slit lamp. Three consecutive photographs were taken for each eye. The first one recorded abnormalities of the anterior segment, such as corneal opacity, iris atrophy, dilated pupil, and glaucomatous fleck. The second one logged the turbidity of crystalline lens. The third one recorded the anterior chamber depth around the limbus on the temporal side using an illuminated slit lamp, with which light cast a clear line on the iris. If a pterygium was found on the temporal side, the nasal side was photographed. If a clear photograph could not be obtained due to pterygium on both sides or the subject’s non-cooperation, the community ophthalmologist would make a rough judgment on the depth of the peripheral anterior chamber based on the slit-lamp examination.

Digital fundus photographs were taken using a digital nonmydriatic fundus camera (CanonCR-DGi, Japan), and 45° of field was captured. Centered by optic disc and macula, two fundus photographs were taken. If the pupil was small, refractive media was not clear, or the subject was not cooperative, the photographs taken would be unsatisfactory. After the subjects with peripheral anterior chamber depth less than 1/4 corneal thickness (CT) were excluded, the other subjects’ pupils were dilated with tropicamide/phenylephrine eye drops, and then photographs were taken. If the photographs were still not clear, “vague fundus” was recorded.

All the data that had been collected remotely were transmitted to Shanghai Eye Disease Prevention and Treatment Center, and the film-reading doctors used Van Herick method to evaluate the peripheral anterior chamber depth. The doctors used Microsoft Paint to measure the depth of the peripheral anterior chamber and the corresponding CT in the anterior segment photograph, and then calculated the ratio of the anterior chamber depth to the CT. The doctors also used Microsoft Paint to measure the vertical diameters of the optic cup and optic disk, and then calculated the vertical cup to disk ratio (VCDR). When the optic disk boundary was determined, the parapapillary atrophy and the sclera ring of Elschnig should be excluded. The margins of the cup were defined as the point of maximum inflection of the vessels crossing the neuroretinal rim. Disk hemorrhage, notch, and other abnormal characteristics on the fundus photograph were recorded.

Gonioscopy was performed with a Goldman one-mirror lens (HaggStreit, Bern, Switzerland) at 16 × magnification using a 1-mm-long slit lamp with low ambient illumination to prevent light from irradiating the pupil area. A vertically irradiated light beam was used for observing both the superior and the inferior anterior chamber angles and horizontally for the nasal and temporal quadrants. Anterior chamber angle was first evaluated statically, and then dynamic examination with the lens was performed. The details of the anterior chamber angles were recorded with the Spaeth grading system.

All the glaucoma suspects were asked to test the visual field in the tertiary eye hospital. SITA-FAST 30–2 mode white-on-white automated perimetry (Humphrey 720, Carl Zeiss, Meditec. Inc., CA, USA) was performed with refractive correction. If the perimetry had low reliability, training on the check method for the suspects was conducted. If visual field defects were found, the perimetry was rechecked 1 h later.

RNFL thickness was measured using the spectral domain Cirrus HD-OCT (Carl Zeiss, Meditec. Inc., CA, USA). Each eye was measured three times, and the one with the strongest signal was selected for analysis.

### Diagnostic definitions

Glaucoma suspects were identified according to any of the following signs: VCDR >0.5 in either eye, VCDR asymmetry ≥0.2, or a neuroretinal rim width reduced to <0.1 CDR (between 11 and 1 o’clock or 5 and 7 o’clock), optic disk hemorrhage, notch in the optic disc rim or obvious RNFL defects on the superior or inferior temporal near the disc in the fundus photograph, and IOP ≥21 mmHg.

A glaucomatous visual field defect was defined as a glaucoma hemifield test result displayed as “outside normal limits” combined with four or more adjacent scotomas not crossing the horizontal meridian on the pattern deviation plot (*P* < 5 %) [8].

Glaucoma cases were diagnosed using the ISGEO criteria. Glaucoma was identified in accordance with three levels of evidence. The highest level of evidence requires optic disc and visual field abnormalities (VCDR or asymmetry in the 97.5th percentile or more for the normal population with a definite glaucomatous visual field defect). In the second level, if a visual field test result is not satisfactory, a severely damaged optic disc (VCDR or asymmetry in the 99.5th percentile or more for the normal population) would be sufficient to make the diagnosis. In the third level, if the optic disc could not be examined because of severe media opacity, subjects who are blind (BCVA < 3/60) combined with either an IOP >99.5th percentile or definite glaucoma medical records, such as filtration surgery, were diagnosed as glaucoma. The division of glaucoma into primary angle-closure glaucoma (PACG) and POAG was based on the gonioscopic finding of a narrow angle (a synonym of occludable angle, where the posterior, usually pigmented, trabecular meshwork is not visible for 270° or more during a static examination). With secondary factors excluded, primary glaucoma can be diagnosed. Primary glaucoma is divided into POAG (open-angle) and PACG (angle-closure) based on the gonioscopy result.

Blindness was defined as BCVA of worse than 20/400 or visual field constriction to less than 10° of fixation in the better eye.

### Statistical analysis

A database was established with EpiData 3.0 (EpiData Association, Odense, Denmark). Statistical analysis was performed using SAS version 9.1.3 (SAS Inc., NC, USA). Data were presented as prevalence and odds ratios (OR) with the corresponding 95 % confidence interval (CI). The prevalence of POAG was adjusted by the standards ratio of population in Pudong New District,and the age- and gender-specific prevalence of POAG and their 95 % CI were calculated. Categorical variables were compared between groups using chi-square tests. This study first performed univariate logistic regression analysis on the factors influencing POAG, and then performed multivariate logistic regression analysis on the factors that were statistically significant for the difference using the step-back technique (*α*_in_ = 0.1; *α*_out_ = 0.1). A value of *P* <0.05 was defined as statistically significant.

## Results

A total of 2528 subjects of the enumerated 3146 adults participated in the study with valid data, giving a response rate of 80.36 %, while 56 subjects were excluded because of incomplete records in one or more respects.

Among 2528 subjects with complete data, 1068 (42.25 %) were men and 1460 (57.75 %) were women. Women were more likely to participate than men (*P* < 0.001). The mean age of the population with responses was 63.54 ± 8.83 years (in the range of 50–106 years). People at both ends of the age distribution were less likely to participate in this study. Table 1 presents the details of the population in this study.

**Table 1:** Demographic characteristics of enumerated and examined subjects*

Age	Enumerate no. (%)			Examined no. (%)			% Examination response rate
	Male	Female	All (%)	Male	Female	All (%)	
50-59	651	641	1292 (41.07)	371	611	982 (38.84)	76.01
60-69	553	547	1100 (34.97)	413	510	923 (36.51)	83.91
70-79	247	320	567 (18.02)	230	256	486 (19.22)	85.71
≥80	75	112	187 (5.94)	54	83	137 (5.42)	73.26
Total	1526 (48.51)	1620 (51.49)	3146 (100)	1068 (42.25)	1460 (57.75)	2528 (100)	80.36

^*^56 subjects with incomplete data were excluded from the analysis

The IOP distribution of normal population was calculated by excluding data of subjects with definite glaucomatous visual field defects or angle closure. The mean values and standard deviations of IOP on the right eye of normal subjects were 16.03 ± 3.33 mmHg, with the male being 16.14 ± 3.40 mmHg and the female being 15.94 ± 3.28 mmHg. The sex difference was not statistically significant (*P* = 0.13). The median, 97.5th percentile, and 99.5th percentile of IOP on the right eye of normal subjects were16 mmHg, 23 mmHg, and 26 mmHg, respectively (Table 2). The 99.5th percentile (26 mmHg) for IOP was used as the cutoff value for category 3 glaucoma diagnosis.

**Table 2:** Distribution of intraocular pressure and VCDR in normal subjects

	Intraocular pressure^a^		Vertical cup-to-disc ratio^b^		
	Right (mmHg, 95 % CI)	Left (mmHg, 95 % CI)	Right (95 % CI)	Left (95 % CI)	Asymmetry
Percentile	2407	2406	2414	2419	2390
0.5th	8 (8–9)	9 (8–9)	0.1 (0.1–0.10)	0.1 (0.1–0.1)	0 (0–0)
2.5th	10 (10–10)	11 (10–11)	0.11 (0.1–0.13)	0.12 (0.1–0.13)	0 (0–0)
50th	16 (16–16)	16 (16–16)	0.34 (0.33–0.34)	0.34 (0.34–0.35)	0.05 (0.05–0.05)
97.5th	23 (23–24)	23 (23–24)	0.55 (0.54–0.57)	0.58 (0.56–0.59)	0.17 (0.16–0.18)
99.5th	26 (25–27)	26.97 (26–27)	0.64 (0.6–0.66)	0.66 (0.63–0.67)	0.24 (0.2–0.27)

^a^Defined as available IOP by excluding eyes with definitive glaucomatous field defects or angle closure

^b^Defined as available VCDR by excluding eyes with definitive glaucomatous field defects

The distribution of VCDR was calculated by excluding eyes with definite glaucomatous visual field defects. If a VCDR could not be determined, data missing were recorded. The absolute value of the determinable VCDR difference in both eyes was used as the asymmetric value of the VCDR of both eyes. The median, 97.5th percentile, and 99.5th percentile of the VCDR on the right eye of normal subjects were 0.34, 0.55, and 0.64, respectively (Table 2). The median, 97.5th percentile, and 99.5th percentile of the asymmetry value of the VCDR were 0.05, 0.17, and 0.24, respectively. The 97.5th and 99.5th percentiles of the VCDR on the left or right eye and those for the asymmetry value of the VCDR were used as the cutoff value for category 1 and category 2 glaucoma diagnoses, respectively.

According to the ISGEO criteria, 72 POAG cases were detected in this study, with a male-to-female ratio of 37:35. Their ages ranged from 51 to 99, and the median of the age was 65. According to the criteria for category 1, category 2, and category 3 glaucoma diagnoses, 49, 19, and 4 people were diagnosed with POAG, respectively. Among 72 POAG, 9 (12.5 %) were blind in one eye, 8 (11.11 %) had already been diagnosed with POAG before this investigation, and 50 people (69.44 %) had IOP lower than 21 mmHg (Table 3).

**Table 3:** Characteristics of POAG

Diagnosis	No.	M:F ratio	Median age (Range)	Diagnostic category			Blind in at least one eye (%)	Previously diagnosed (%)
				1	2	3		
POAG with normal IOP	50	26:24	63 (53–99)	36	14	0	4 (8)	2 (4)
POAG with high IOP	22	11:11	66 (51–86)	13	5	4	5 (22.73)	6 (27.27)
Total POAG	72	37:35	65 (51–99)	49	19	4	9 (12.5)	8 (11.11)

The crude prevalence rate of POAG in this study was 2.85 % (95 % CI: 2.2 %–3.5 %). Table 4 shows the information about the patients of different ages, sexes, and diagnostic categories. The prevalence rates in different sex groups were statistically insignificant (*χ*^2^ = 2.54; *P* = 0.11). In contrast, as the age increased, the prevalence rates in different age groups were statistically significant (*χ*^2^ = 5.43; *P* = 0.02). After the age and sex were standardized according to 2011 Shanghai Pudong New District Census, the prevalence rate of POAG was 2.80 % (95 % CI: 2.78 %–2.83 %).

**Table 4:** Prevalence of POAG by age and sex

Age (yrs)	Male					Female					All	
	No.	Diagnostic category			Prevalence (95 % CI)	No.	Diagnostic category			Prevalence (95 % CI)	No.	Prevalence (95 % CI)
		1	2	3			1	2	3			
50-59	371	4	5	0	2.43 (0.86–3.99)	611	5	5	0	1.64 (0.63–2.64)	982	1.93 (1.07–2.80)
60-69	413	14	3	1	4.36 (2.39–6.33)	510	7	3	0	1.96 (0.76–3.16)	923	3.03 (1.93–4.14)
70-79	230	7	1	1	3.91 (1.41–6.42)	256	9	1	1	4.30 (1.81–6.78)	486	4.12 (2.35–5.88)
≥80	54	1	0	0	1.85 (−1.74–5.45)	83	2	1	1	4.82 (0.21–9.43)	137	3.65 (0.51–6.79)
Total	1068	26	9	2	3.46 (2.37–4.56)	1460	23	10	2	2.40 (1.61–3.18)	2528	2.85 (2.20–3.50)

Univariate regression analysis on each variable that might be related to POAG showed that independent risk factors for POAG included age, family history of glaucoma, IOP, myopia, and hypertension (Table 5). Then, multivariate logistic regression analysis was performed between the statistically significant factors and POAG, and age group of 70–79 (*P* = 0.01, OR and 95 % CI: 2.38 (1.21–4.67)), family history of glaucoma (*P* < 0.0001, OR and 95 % CI: 15.99 (4.86–52.57)), IOP >21 mmHg (*P* < 0.0001, OR and 95 % CI: 7.52 (4.29–13.16)), myopia (*P* = 0.05, OR and 95 % CI: 1.94 (1.01–3.75)), and hypertension (*P* = 0.09, OR and 95 % CI: 1.53 (0.93–2.50)) were identified as significant independent risk factors.

**Table 5:** Univariate and multivariate logistic regression analysis of risk factors for POAG

Risk factor		POAG	Non-POAG	Univariate		Multivariate	
				*p* value	OR (95 % CI)	*p* value	OR (95 % CI)
Sex	Male	37	1031		1	-	-
	Female	35	1425	0.11	0.68 (0.43–1.09)	-	-
Age	50–59	19	963		1		1
	60–69	28	895	0.13	1.59 (0.88–2.86)	0.25	1.44 (0.78–2.65)
	70–79	20	466	0.02	2.18 (1.15–4.12)	0.01	2.38 (1.21–4.67)
	≥80	5	132	0.20	1.92 (0.71–5.23)	0.13	2.22 (0.79–6.19)
Education level	Primary	6	274		1	-	-
	Secondary	33	1301	0.74	1.16 (0.48–2.79)	-	-
	Higher	33	879	0.23	1.71 (0.71–4.14)	-	-
Family history	No	60	2445		1		1
	Yes	12	11	<0.0001	16.59 (5.61–49.07)	<0.0001	15.99 (4.86–52.57)
IOP	≤21 mmHg	50	2313		1		1
	>21 mmHg	22	143	<0.0001	7.12 (4.19–12.08)	<0.0001	7.52 (4.29–13.16)
Diabetes	No	62	2189		1	-	-
	Yes	10	267	0.42	1.32 (0.67–2.61)	-	-
Myopia	No	60	2229		1		1
	Yes	12	227	0.04	1.96 (1.04–3.71)	0.05	1.94 (1.01–3.75)
Hyperopia	No	67	2269		1	-	-
	Yes	5	187	0.83	0.91 (0.36–2.27)	-	-
Cataract	No	60	2177		1	-	-
	Yes	12	279	0.17	1.56 (0.83–2.94)	-	-
Hypertension	No	35	1565		1		1
	Yes	37	891	0.01	1.86 (1.16–2.97)	0.09	1.53 (0.93–2.50)

## Discussion

This is the first population-based POAG screening conducted in Shanghai. Using the digital remote screening model, general eye examinations such as IOP testing and retinal photography were performed. POAG suspects were followed up and diagnosed by Shanghai Eye Disease Prevention and Treatment Center based on the ISGEO criteria used worldwide [4–10].

In this study, telemedical screening mode and reexamination diagnosis were used. Information was first collected and analyzed remotely, including IOP, anterior chamber angle depth, vertical cup-to-disc ratio of optic nerve, rim notch, hemorrhage on or around optic nerve head, and vascular changes or visible RNFL defects. An appointment was then made at a tertiary hospital for suspected subjects to accept standard examinations for glaucoma diagnosis. A screening procedure could be done by a team of five to six screening personnel with optometrists and community primary eye care physician without ophthalmology doctors in the selected committees. Here 92.2 % attendants were satisfied with the screening. Importantly, many other retinal disorders, such as diabetic retinopathy and macular degeneration, were detected in the screening. Compared to other epidemiological investigations, this screening model can reduce the ophthalmologist’s workload [5–9], as required considering the deficiency of ophthalmologists and a large elderly population in China, and has the capability of extending glaucoma screening and other elderly eye disease screening in the community [11]. In addition, 97.5th and 99.5th percentiles for normal VCDR was found to be lower than previously reported by other researchers. The reason might be that VCDR obtained directly from fundus examination by ophthalmologists was usually higher than that obtained from fundus photographs and Microsoft Paint software used. In this study, the vertical diameters of the cup and disc were precisely measured from the fundus photographs while other groups used the standard photo comparison method [7].

The unadjusted prevalence rate of POAG in this survey was 2.85 % (95 % CI, 2.20–3.50). When compared with other studies conducted in different countries, the prevalence rate of POAG was lower in eastern China than in black populations (3.1 %–7 %) [12–14] and other Asian populations (3.5 %–4 %) [15–17], and was similar to the rate reported in Australia (95 % CI: 2.5 %–3.6 %) [18]. The rate, however, was higher than that reported in other white populations (0.8 %–2.51 %) [19–22], Hispanic populations (95 % CI: 1.58 %–2.36 %) [23], and Mongolia (0.5 %) [24]. Genetic profiles, environmental factors, and/or diagnostic methods used for defining POAG would seem to cause such variations. When different regions in China were considered, the prevalence rate in this survey was higher than that in Beijing (2.6 %, 95 % CI: 2.1 %–3.0 %) [5], urban Guangzhou (2.1 %, 95 % CI: 1.4 %–2.8 %) [8], rural Inner Mongolia (1.4 %, 95 % CI: 0.79 %–2.02 %) [4], Bai Nationality in Yunnan (1.0 %, 95 % CI: 0.6 %–1.6 %) [9], and rural Harbin (0.71 %, 95 % CI: 0.47–0.93) [7]. These differences might be attributed to geographical and ethnic differences, since China is geographically the third largest country with 56 distinct ethnic groups. In addition, different instruments and approaches used in these studies might contribute to the differences.

A high prevalence of normal-tension glaucoma (NTG) has been reported in Japan [16], Korea [17], as well as Liwan District in Guangzhou [8], China. In Huamu community, 50 of the 72 POAG subjects (69.44 %) had IOP less than 21 mmHg. The average IOP in untreated POAG patients with normal pressure and high pressure was 15.68 ± 2.27 mmHg and 23.06 ± 2.88 mmHg, respectively. The average IOP on the right and left eye of the POAG patients with normal IOP was slightly lower than that in normal subjects. Therefore, the diagnosis of POAG cannot focus only on IOP measurement in case POAG patients with normal IOP are neglected. Many studies showed that IOP fluctuated during the daytime for both healthy individuals and POAG patients, and the fluctuation could influence the diagnostic and prognostic evaluation of the glaucomatous disease [25–28]. Only one IOP test was conducted in the daytime during the screening, which could not reflect all IOP values of the tested subjects in other time points and might generate bias on a higher rate of NTG. Therefore, subjects cannot be diagnosed as NTG according to the random IOP value obtained during the day.

As noted earlier, the majority of the POAG patients were not detected [4–9, 16, 17]. Before the screening, 88.89 % of POAG patients were undiagnosed and only 4 % POAG patients with normal IOP had been diagnosed. Glaucoma showed no symptoms, and a majority of patients had normal IOP at an early stage. Residents aged 50 and older in Huamu community have less knowledge on glaucoma. In addition, optometrists and primary care physicians in Huamu Community Health Service Center may lack clinical experience on glaucoma diagnosis. Owing to these reasons, the majority of the POAG patients could not be diagnosed before this screening. Nine out of 72 subjects were blind in one eye and most had obvious vision loss when diagnosed in this study, suggesting that more efforts are needed on early screening of POAG and public education on glaucoma for prevention of blindness in Shanghai.

Univariate and multivariate logistic regression analyses showed that age, family history of glaucoma, IOP, myopia, and hypertension were all risk factors related to POAG.

A number of studies reported that the glaucoma prevalence rate increased with age [4–9]. However, a significant difference was only found between the age group from 70 to 79 and that from 50 to 59. The reason for the bias might be that the attendances in the highest age group were lower than those in any other age group due to poorer mobility and health conditions, with an attendance rate of only 73.26 %.

Several studies have demonstrated that the family history of glaucoma is associated with the presence and severity of POAG [29, 30]. The Utah Population Database (UPDB) study found a significantly higher prevalence rate of glaucoma in both first- and second-generation relatives and the population attributable risk of the genetic factors in this population was 0.20. It was concluded that the family history for glaucoma played an important role in the presence of POAG, although the factor might have been underestimated in this study due to lack of knowledge or unawareness of the disease for the tested subjects. Therefore, screening the first- and second-generation relatives will be an effective way to detect glaucoma in a population.

As reported earlier, IOP is another important risk factor of POAG. The present study found 73 of all attendants (2.89 %) having high IOP without any sign of vision impairment. Many studies showed that IOP was a good predictor for the onset of POAG, and demonstrated that topical ocular hypotensive medication was effective in delaying or preventing the onset of POAG in individuals with elevated IOP [31, 32]. Therefore, individuals with ocular hypertension should be followed up at regular intervals or considered with initiating interventions.

Increasing studies have indicated that high myopia is important in the pathogenesis of glaucoma, especially for POAG [7, 16, 33], and the indication is consistent with the present findings. Population-attributable risk estimates are best used to prioritize medical and public health interventions based on the magnitude of the potential effects of a risk factor on the disease outcome in the community. It is important to investigate the factors of refractive errors associated with glaucoma in longitudinal studies. Subjects with high myopia should be screened for glaucoma at shorter intervals.

The present study found that hypertension was also a risk factor for POAG, which had not been reported in any other population-based glaucoma studies [34–36].

The present study had certain limitations. Not all the attendants received visual field examination, thereby neglecting the diagnosis of some patients with light damage of the optic nerve and hence inducing an underestimate of the prevalence rate of POAG. Noncontact tonometer, which was not a standard tool for screening or diagnosing glaucoma, was used for both preliminary screening and reexamination. It might have lead to an overestimation of IOPs and issues with variation with higher or lower corneal thickness. In addition, lack of attendance might also introduce bias. In the present study, the male subjects, especially those aged 50–60, were underrepresented in the sample with the participation rate of only 52.32 %. The reason might be that males aged 50–60 were more socioeconomically active or less aware of eye diseases. Therefore, public education on glaucoma should be the focus in the future.

## Conclusion

Pudong New District, Shanghai, has more POAG patients compared with other places in China. Among them, the majority have normal IOP, and most of the POAG patients have not been diagnosed or treated before. Therefore, more efforts are required on early screening and education on POAG in communities, especially in a POAG high-risk population.

## Abbreviations

POAG: Primary open-angle glaucoma
IOP: Intraocular pressure
RNFL: Retinal nerve fiber layer
OCT: Optical coherence tomography
ISGEO: International Society for Geographic and Epidemiological Ophthalmology
ID: Identity
LogMAR: Minimum angle of resolution
BCVA: Best corrected visual acuity
CT: Corneal thickness
VCDR: Vertical cup to disc ratio
PACG: Primary angle-closure glaucoma
OR: Odds ratios
CI: Confidence interval
NTG: Normal-tension glaucoma
UPDB: Utah Population Database
